# The Independent Association of Plasma and Red Blood Cell Zinc Concentrations with Long-Term Outcomes of Hospitalized Patients

**DOI:** 10.1016/j.cdnut.2023.100062

**Published:** 2023-03-02

**Authors:** Stefan Rodic, Christopher McCudden, Carl van Walraven

**Affiliations:** 1 Department of Medicine, University of Ottawa, Ottawa, Canada; 2 Department of Pathology and Laboratory Medicine, University of Ottawa, Ottawa, Canada; 3 Division of Biochemistry, The Ottawa Hospital and The Eastern Ontario Regional Laboratory Association, Ottawa, Canada; 4 Department of Medicine and Epidemiology and Community Medicine, University of Ottawa, Ottawa, Canada; 5 Ottawa Hospital Research Institute and ICES (formerly Institute for Clinical Evaluative Sciences), Ottawa, Canada

**Keywords:** zinc, hospital, association, regression, survival

## Abstract

**Background:**

Plasma and RBC zinc values are unrelated in hospitalized patients. The independent association of these values with important patient outcomes is unknown.

**Objectives:**

Measure the independent association of plasma and RBC zinc with outcomes in hospitalized patients.

**Methods:**

Plasma and RBC zinc concentrations were prospectively measured within 48 h of hospitalization in consenting patients. Data were linked deterministically with population-based health administrative data to measure each association of zinc measures with 2 outcomes (time to death from any cause and likelihood of death or urgent readmission to hospital within 30-d of discharge) after adjusting for validated outcome risk scores.

**Results:**

In total, 250 people admitted to medical services were studied. Patients were ill with a 1-y baseline expected death risk (IQR) of 19.9% (6.3%–37.2%). The observed 1-y and 2-y all-cause death risks were 24.5% (95% CI: 19.6%, 30.3%) and 33.2% (95% CI: 27.3%, 39.9%), respectively. Death risk increased significantly as plasma zinc concentrations decreased (*P* = 0.0001). This association persisted even after adjusting for the baseline expected death risk (*P* = 0.02) with every 2-μmol/L decrease in plasma zinc concentrations being independently associated with, on average, a 35% increase in the death risk. RBC zinc concentrations were not associated with the death risk. Neither plasma nor RBC zinc concentrations were significantly associated with the 30-d death or urgent readmission rate.

**Conclusions:**

Plasma, but not RBC, zinc concentrations are independently associated with the all-cause death risk in hospitalized medical patients. Further study is required to determine whether this association is causal and identify its potential causal pathways. *Curr Dev Nutr* 2023;x:xx.

## Introduction

Zinc is a cofactor in a wide variety of enzymatic pathways. Zinc deficiency in humans can cause slow growth, impaired immunity, poor wound healing, and various skin disorders [1,2]. Low plasma or serum zinc concentrations have been identified in more than half of hospitalized patients, with a higher prevalence in critical care units and among patients with heart failure, cirrhosis, and chronic kidney disease [[3], [4], [5], [6], [7], [8], [9]].

Multiple studies have explored the association between patient zinc concentrations and outcomes in hospitalized patients [10]. Serum/plasma zinc has been primarily associated with poor outcomes in different disease contexts, including in patients with heart failure, sepsis, or myocardial infarction. Patient survival was the most common outcome with time horizons ranging from the hospitalization (the most common) to 5 y. Seven of the 11 studies found a significant association between low zinc concentrations and an increased death risk.

However, the literature exploring zinc concentrations and outcomes is limited. Most studies had <125 patients, used unspecified sampling methods, were limited to critically ill patients, measured zinc concentrations at undefined time points of the admission, and rarely considered potential confounding factors. This latter issue is especially important because low zinc concentrations might be a biomarker of severe illness, which is supported by the observation that acute disease processes—such as acute myocardial infarctions or surgery—can acutely lower serum/plasma zinc [11]. Adequate adjustment for important potential confounders in the association of zinc and outcomes is essential to gauge its role as a potential therapy.

Almost all studies examining the association between zinc and outcomes have measured serum or plasma concentrations. However, RBC zinc concentrations can also be measured. Because the RBC zinc concentration is an intracellular measure, it may be less sensitive to short-term changes in health status that are known to acutely lower serum/plasma zinc concentrations [11]. We know that RBC and serum/plasma zinc concentrations are essentially independent of each other [12]. However, we do not know whether serum/plasma or RBC zinc has a stronger independent association with important outcomes for hospitalized patients.

In this study, we measured the association of both plasma and RBC zinc concentrations in a broad group of hospitalized patients with 2 important outcomes—time to death and likelihood of the 30-d death or urgent hospital readmission rate—after controlling for validated risk quantifiers of both outcomes.

## Methods

### Study setting, design, and data set descriptions

The study took place at The Ottawa Hospital, a 1000-bed teaching hospital that is the tertiary referral center in our region of 1.3 million people (2016 census). The study was approved by our research ethics board (File 20200349).

This study anonymously linked primarily collected data to population-based health administrative data for Ontario housed at ICES (formerly Institute for Clinical Evaluative Sciences). ICES is a publicly funded and independent research organization. We linked data deterministically through encrypted health card numbers to the following 2 data sets to retrieve information required for the study’s analysis: *1*) Discharge Abstract Database, which captures all Ontario hospitalizations recording admission and discharge dates, diagnoses, and procedures; and *2*) Registered Persons Database (RPDB), which records the death date (including those occurring outside of the province) of all Ontarians and the date of the latest encounter people had with the health care system.

We prospectively enrolled patients hospitalized under a medical service within 48 h of admission between September and December 2020. Patients were older than 17 y and admitted to a variety of services such as general internal medicine, cardiology, hematology, medical oncology, or nephrology. We did not enroll surgical patients to avoid the confounding effect of recent surgery on acutely lowering plasma zinc concentrations [11]. Candidate patients were identified daily using our hospital’s electronic medical record. Exclusion criteria included the following: inability to provide informed consent owing to dementia, delirium, or somnolence; and active infection with severe acute respiratory syndrome coronavirus 2 infection (for infection control reasons).

### Study patients and sampling

The study sample size was determined by the maximum grant amount allocated per project by our academic organization ($90,000) and the cost of zinc testing (which was estimated to be $270 per patient, including courier charges). After deducting $5000 for incidental costs, we had enough money to recruit 315 patients. After exclusions (listed in the Results), the study included 250 patients. We considered this sample adequate based on the formulas from Hsieh et al. [13] implemented by the powerMediation package in the R software, which found that a sample size of 250 would have a power of 85% to detect an OR of 1.5 for 1 SD decrease in serum zinc concentrations and death risk and 99.9% to detect an OR of 2.0 for 1 SD decrease in serum zinc concentrations and death risk.

### Zinc sampling and processing

All blood samples were collected by phlebotomists within 48 h of admission on the morning after recruitment to control for diurnal variation in serum zinc concentrations. Plasma and RBC zinc samples were collected in 2 separate metal-free royal blue trace element collection tubes (BD vacutainers; catalog #368380) and were frozen within 1 h of collection. All zinc concentration testing took place at the London Health Sciences Centre, in London, Ontario, using high resolution sector field ICP-MS (HR-SF-ICP-MS). Serum zinc and plasma zinc concentrations are regarded as essentially equivalent measures [14]. We used plasma zinc concentrations because the reference laboratory measuring all heavy metals required the use of plasma for all heavy metal measures (to consider potential contamination procoagulants may have on the measure of some heavy metals other than zinc). We excluded samples that were clotted or contained insufficient quantity of blood for the analysis. The standard adult normal reference ranges for plasma zinc and RBC zinc were 9.4–15 and 138–230 μmol/L, respectively, and was determined by the reference laboratory using a nonoccupationally exposed reference population.

Zinc concentrations were summarized in a data set having patient health card number as the unique identifier. This data set was encrypted and transferred electronically to ICES, where patient health card numbers were encrypted to permit linkage with population-based administrative data sets.

### Outcomes

The primary outcome was the time to death of any cause. This was determined by linking to RPDB to determine if, and when, patients died. The secondary outcome was 30-d death or urgent readmission rates after discharge from the index hospitalization. This was determined by linking to 2 data sets: *1*) RPDB, to determine whether patients died of any cause within 30 d of discharge from hospital and *2*) Discharge Abstract Database, to determine whether patients recorded a nonelective (that is, unplanned) admission to hospital within 30 d of discharge from the hospital.

### Covariates

The primary covariate was the hospitalized-patient 1-y mortality risk (HOMR) score [15]. This score quantifies the expected 1-y risk of all-cause death using health administrative data. It combines values for 12 covariables regarding patient demographics (such as age, sex, and living status), health burden (such as Charlson comorbidity index score, home oxygen use, and both emergency department and hospital utilization in the previous year), and hospitalization factors (such as admission urgency, initial need for critical care, hospital readmission status, and diagnostic risk score). The Charlson comorbidity score, which gauges the independent influence of chronic medical conditions on mortality [16], was calculated using updated weights [17] applied to coded diagnoses in administrative data [18] with a 1-y lookback period. The HOMR is highly discriminative (*c*-statistic, 0.89) and well calibrated for the 1-y all-cause death risk [15]. It has been externally validated using administrative data [19] and primary data [20]. Death risk increases as the HOMR score increases.

The secondary covariate was the length of stay, acuity of admission, comorbidities, and emergency department use (LACE) index [21]. This model quantifies risk of death or unplanned readmission to hospital within 30 d of hospital discharge. Its parameters include index hospitalization length of stay and acuity, patient comorbidity, and the number of emergency department visits in the previous 6 mo. The LACE index is moderately discriminating (*c*-statistic, 0.68) and is well calibrated [21]. It has been externally validated several times [[21], [22], [23], [24]]. Risk of death or unplanned readmission increases as the LACE score increases.

### Analysis

All analyses were conducted using SAS 9.4. Survival was summarized with Kaplan–Meier curves for the entire cohort and stratified by the median plasma zinc and RBC zinc concentrations and HOMR score. Strata-specific survival was compared using the log-rank statistic. We determined nonlinear associations between zinc measures and the HOMR score using linear regression (PROC REG) invoking an algorithm from the study by Sauerbrei et al. [25] to identify best-fitting fractional polynomials. Proportional hazards modeling (PROC PHREG) was used to measure the association of both zinc measures with the time to patient death. Patient observation started on the date they were enrolled into the study and ended at death or the date of their last encounter with a health care provider. Both unadjusted and adjusted (for HOMR scores) models were created.

In secondary analyses, we used logistic regression to separately measure the association of plasma and RBC zinc concentrations with the likelihood of death or urgent readmission within 30 d of discharge. This analysis excluded the 9 people who died in hospital because they, by definition, cannot die or be readmitted to hospital urgently after discharge. We created both unadjusted and adjusted (for the LACE index) models using fractional polynomials to identify nonlinear associations. Finally, we explored whether associations between either zinc measure with outcomes varied by patient age or sex by adding interaction terms to the respective models.

## Results

A total of 314 patients were prospectively recruited into the study (Supplemental Figure 1). Of the 314, 25 patients did not have blood samples taken within 48 h of admission and were excluded. This left with 289 patients with plasma and RBC zinc testing within the designated period (the morning after recruitment). We excluded 16 patients from the analysis owing to insufficient collected blood volume for RBC zinc testing and another 21 samples were excluded owing to clotting. Finally, 2 patients were excluded because they did not have a valid Ontario health card number required to link to provincial data.

Thus, 250 patients were included in this study (Table 1). The mean age was 65.3 ± 16.5 y, and there was an even sex distribution (46.8% male). More than two-thirds of the patients were admitted to the general internal medicine service. Almost three-quarters of the patients lived independently, whereas approximately a quarter received home nursing assistance. Almost two-thirds of the patients (66.8%) recorded a Charlson comorbidity score of 2 or less. More than a quarter of the patients (26.4%) experienced ≥1 unplanned hospitalization in the previous year and more than half (44.2%) ≥1 emergency room visit. Most patients (54.4%) were primarily admitted for issues that were not classified as cardiovascular, respiratory, or gastrointestinal. Approximately 95% of patients were admitted nonelectively with more than half presenting to hospital by the ambulance. Patients had a median expected 1-y death risk of 19.9% (IQR: 6.3–37.2) and median LACE index of 10 (IQR: 8–12). The mean ± SD plasma and RBC zinc concentrations were 8.5 ± 2.2 and 176.1 ± 36.8 μmol/L, respectively.

**TABLE 1:** Description of the study cohort

Variable	Died during the study		Total (*n* = 250)
	No (*n* = 171)	Yes (*n* = 79)	
Patient factors			
Age (y)	62.4 ± 17.0	71.6 ± 13.5	65.3 ± 16.5
Male	76 (44.4)	41 (51.9)	117 (46.8)
Living status			
Independent	141 (82.5)	46 (58.2)	187 (74.8)
Home nursing	30 (17.5)	32 (40.5)	62 (24.8)
Nursing home	0 (0.0)	≤5 (1.3)	≤5 (0.4)
Charlson score			
0–1	77 (45.0)	15 (19.0)	92 (36.8)
2	55 (32.2)	20 (25.3)	75 (30.0)
3–4	25 (14.6)	16 (20.3)	41 (16.4)
5+	14 (8.2)	28 (35.4)	42 (16.8)
Home oxygen	≤5	5–10	10 (4.0)
Unplanned admissions last year			
0	141 (82.5)	43 (54.4)	184 (73.6)
1	16 (9.4)	19 (24.1)	35 (14.0)
2+	14 (8.2)	17 (21.5)	31 (12.4)
ED visits last year			
0	83 (48.5)	34 (43.0)	117 (46.8)
1	48 (28.1)	18 (22.8)	66 (26.4)
2	17 (9.9)	7 (8.9)	24 (9.6)
3+	23 (13.5)	20 (25.3)	43 (17.2)
Hospitalization factors			
Clinical system			
Cardiovascular	41 (24.0)	19 (24.1)	60 (24.0)
Respiratory	17 (9.9)	11 (13.9)	28 (11.2)
Gastrointestinal	18 (10.5)	8 (10.1)	26 (10.4)
Other	95 (55.6)	41 (51.9)	136 (54.4)
Admission type			
Elective	5–10	≤5	14 (5.6)
Unplanned, no ambulance	71 (41.5)	25 (31.6)	96 (38.4)
Unplanned, ambulance	91 (53.2)	49 (62.0)	140 (56.0)
Unplanned 30-d readmission	16 (9.4)	15 (19.0)	31 (12.4)
HOMR diagnostic score			
<0	51 (29.8)	8 (10.1)	59 (23.6)
0	108 (63.2)	50 (63.3)	158 (63.2)
>0	12 (7.0)	21 (26.6)	33 (13.2)
Admitted to critical care	5–10	≤5	10 (4.0)
ALC during admission	10 (5.8)	8 (10.1)	18 (7.2)
HOMR points	33 (27–38)	41 (37–43)	36 (30–40)
*P*[1-y death]	0.114 (0.033–0.271)	0.428 (0.232–0.507)	0.199 (0.063–0.372)
LACE score	9 (7–12)	12 (9–14)	10 (8–12)
Observation days	707 (670–733)	104 (47–361)	673 (362–721)
Plasma zinc (μmol/L)	8.9 ± 2.2	7.7 ± 2.1	8.5 ± 2.2
RBC zinc (μmol/L)	174.3 ± 35.0	179.9 ± 40.3	176.1 ± 36.8

Values are given as mean ± SD, *n* (%), or median (IQR). ALC, alternate level of care; ED, emergency department; HOMR, hospitalized-patient 1-y mortality risk; LACE, length of stay, acuity of admission, comorbidities, and emergency department use.

**FIGURE 1:**
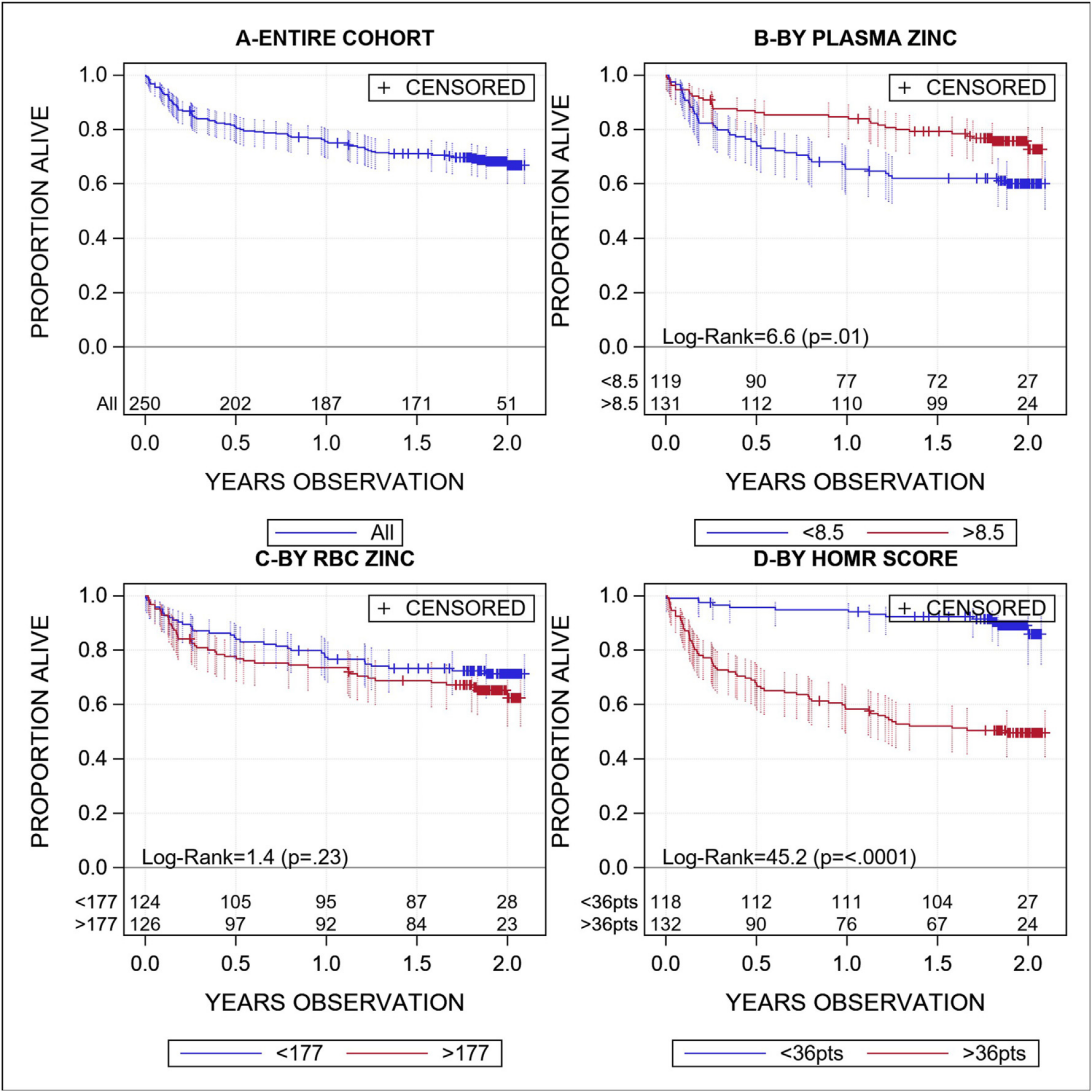
Survival in the entire cohort (A), stratified by zinc concentrations and hospitalized-patient 1-y mortality risk (HOMR) scores. Each plot presents the proportion of the cohort alive (vertical axis) as a function of time (horizontal axis) with 95% CIs. The results are stratified by median values for plasma zinc (B), RBC zinc (C), and HOMR scores (D) [15]. The number of patients observed and significance of difference in strata survival is presented at the bottom of each plot. Plasma and RBC zinc concentrations are measured in micromoles per liter.

The median cohort observation was 673 d (IQR: 362–721). The 1-y and 2-y survival for the entire cohort was 75.5% (95% CI: 69.7, 80.4) and 66.8% (95% CI: 60.1, 72.7), respectively (Figure 1A). Death risk was notably higher during the initial 3 mo of observation, during which time the Kaplan–Meier curve had a notably more negative slope (Figure 1A). Survival was significantly lower for patients with plasma zinc concentrations less than the median value (8.5 μmol/L) (Figure 1B). By contrast, RBC zinc concentrations stratified by the median value of 177 μmol/L was not associated with survival (Figure 1C). The HOMR score was very strongly associated with survival (Figure 1D), with 2-y survival probabilities in those greater than and less than a median HOMR score of 36 points of 85.8% (95% CI: 74.7, 92.3) and 49.5% (95% CI: 40.7, 57.8), respectively.

Compared with patients who were alive, patients who died during the study (Table 1) were older (mean age, 71.6 compared with 62.4 y), were less likely to live independently (58.2% compared with 82.5%), and recorded higher Charlson scores (proportion with score 5+, 35.4% compared with 8.2%), more previous urgent admissions and emergency department visits, and higher HOMR scores (mean 41 compared with 33 points). Moreover, patients who died recorded lower plasma zinc concentrations (7.7 compared with 8.9 μmol/L), but RBC zinc concentrations did not differ notably (179.9 compared with 174.3 μmol/L).

**FIGURE 2:**
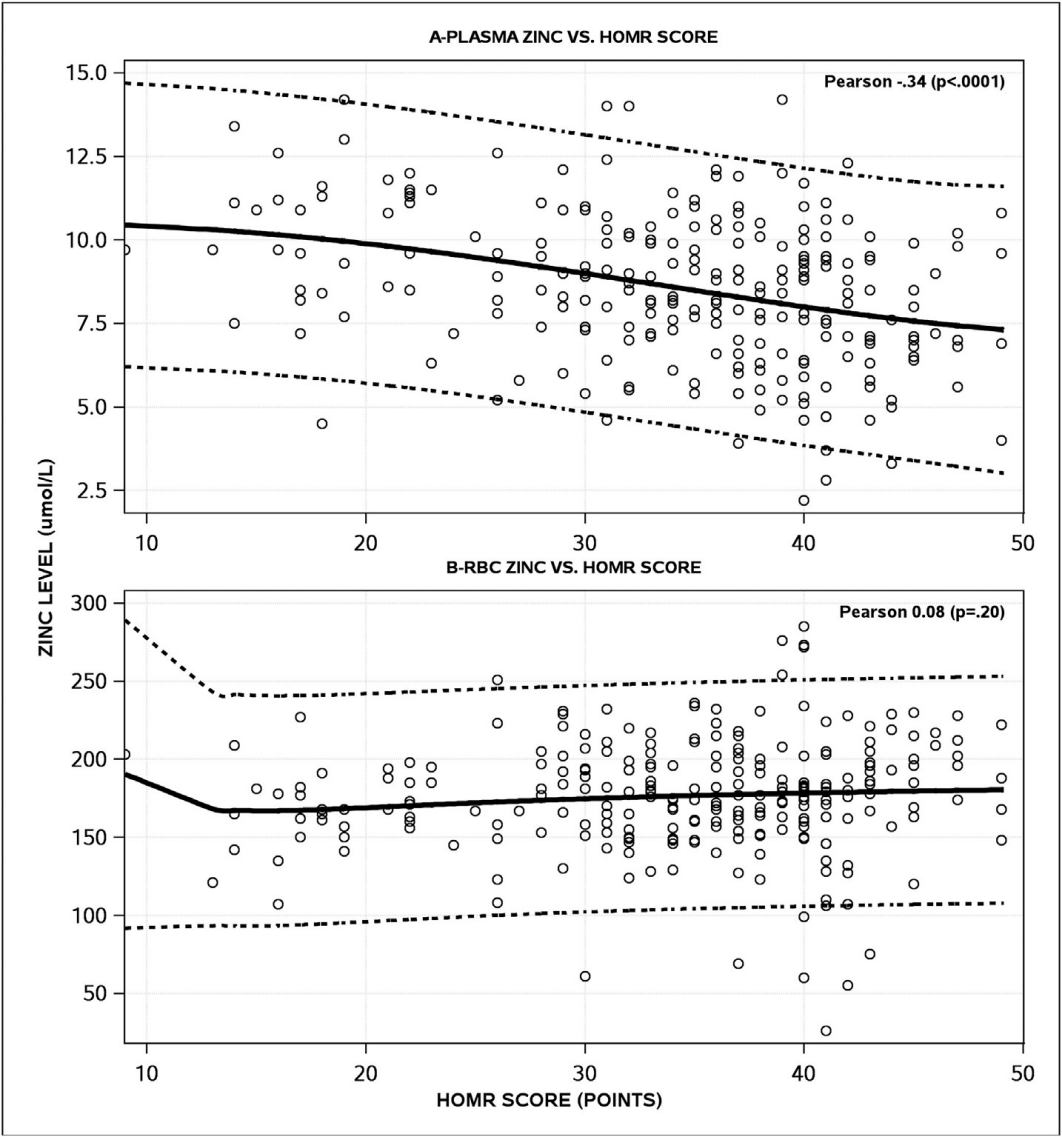
Associations between the zinc concentrations and hospitalized-patient 1-y mortality risk (HOMR) scores. These plots present the correlation of the HOMR score [15] (horizontal axis) with both plasma zinc (A) and RBC zinc (B) concentrations. The Pearson correlation coefficient is presented at the top right of each plot. Nonlinear associations (with 95% CIs) are also presented. Plasma and RBC zinc concentrations are measured in micromoles per liter.

Plasma zinc concentrations were significantly associated with the HOMR score (Figure 2A). As plasma zinc score decreased, the HOMR score—and the expected death risk—increased with a Pearson correlation coefficient of −0.34 (*P* < 0.0001). We also explored nonlinear models, showing that the association between the plasma zinc concentration and HOMR score was slightly nonlinear with a cubic term along with its interaction with a logged plasma zinc term fitting the data the best (Figure 2A). Between the RBC zinc concentrations and HOMR score, we found no linear (Pearson correlation coefficient, 0.08; *P* = 0.2) nor nonlinear associations (Figure 2B).

**FIGURE 3:**
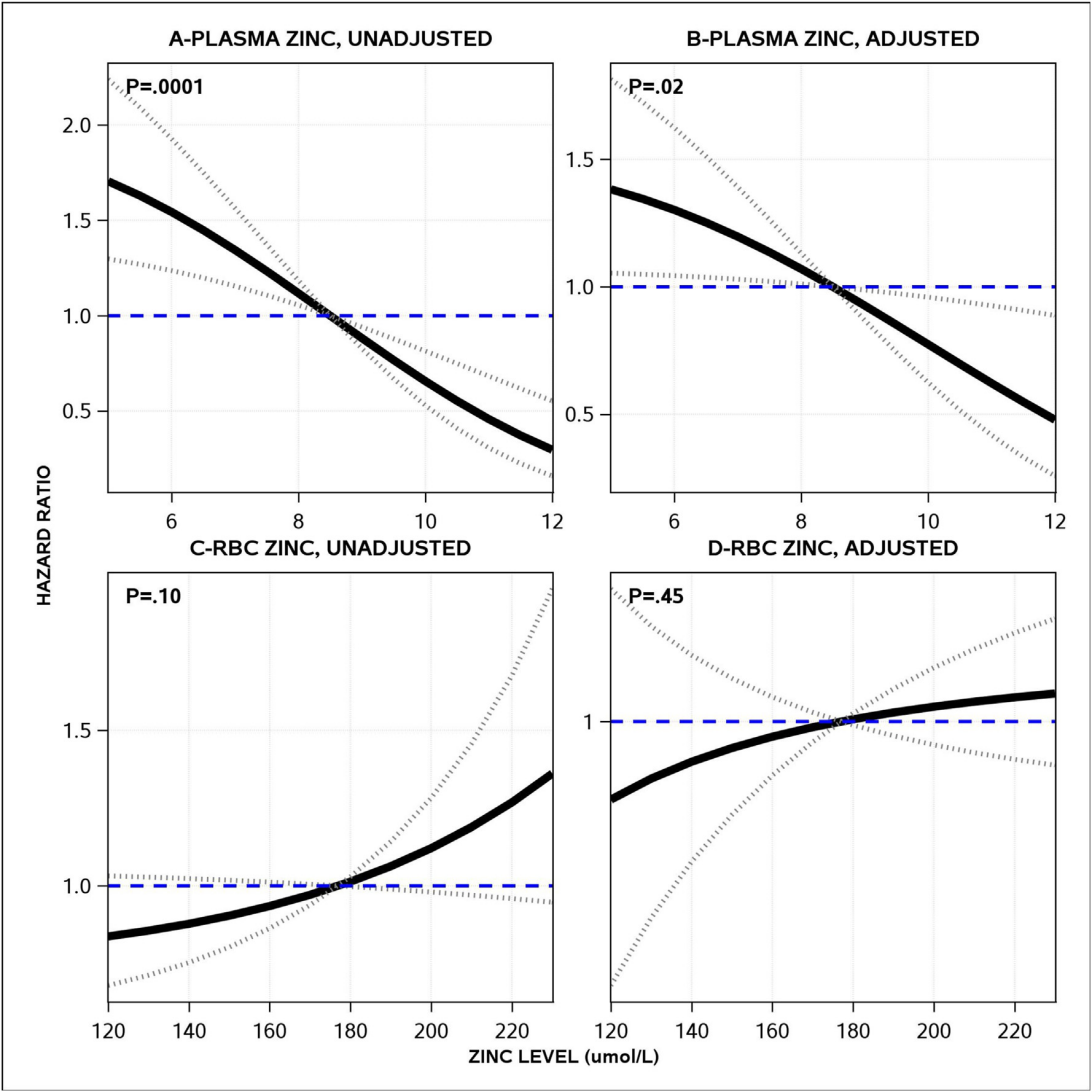
Unadjusted and adjusted associations between the zinc concentrations and death risk. Each plot presents unadjusted and adjusted hazard ratios for time to death (vertical axis) for plasma zinc (A, B) and RBC zinc (C, D) concentrations. Each plot presents hazard ratios relative to the median zinc values in the sample. Hazard ratios exceeding 1 indicate an increased risk of death. The 95% CIs are presented along with the *P* values for the association of zinc concentrations with the death risk. Plasma and RBC zinc concentrations are measured in micromoles per liter.

Plasma and RBC zinc concentrations varied in their relationship with death. Death risk increased significantly as plasma zinc decreased (Figure 3A). This relationship was moderated slightly after adjusting for the HOMR score but remained statistically significant (Figure 3B). On average, every 2-μmol/L decrease in plasma zinc was independently associated with a 35% increase in death risk. Unexpectedly, death risk actually increased—although not significantly (*P* = 0.10)—as RBC zinc concentrations increased (Figure 3C). However, after adjusting for the HOMR score, we found no association between RBC zinc concentrations and death risk (Figure 3D).

Of the 241 people who survived their index hospitalization, 51 (21.2%) died or recorded an urgent hospital readmission rate within the 30 d of discharge. By itself, the plasma zinc concentration was closely—but not significantly—associated with risk of the 30-d death or readmission, with risk increasing as plasma zinc concentrations decreased (Table 2). This association lessened notably after adjusting for LACE score, which was strongly and positively associated with the outcome risk (Table 2). Similarly, we also found a trend toward an unadjusted association between RBC zinc concentrations and 30-d death or urgent readmission rates; however, this unadjusted analysis found that increasing RBC zinc concentrations were associated with an increased outcome risk (Table 2). After adjusting for the LACE score, this trend persisted (Table 2). No significant interactions existed between either age or sex and the association between either plasma or RBC zinc concentrations with either time to death or 30-d death or unplanned readmission rates (Supplemental Table 1).

**TABLE 2:** Association of plasma and RBC zinc concentrations with 30-d death or urgent readmission

Variable	Parameter estimate (SE)	*P*
Plasma zinc, unadjusted		
Intercept	−0.12 (0.62)	0.8508
(Plasma zinc)^0.5^	−0.14 (0.07)	0.0518
Plasma zinc, adjusted		
Intercept	−1.65 (0.79)	0.0362
(LACE score)^2^	0.01 (0)	0.0006
(Plasma zinc)^0.5^	−0.08 (0.08)	0.3097
RBC zinc, unadjusted		
Intercept	−2.05 (0.45)	0
(RBC zinc)^2^	1.23 × 10^−5^ (0)	0.0707
RBC zinc, adjusted		
Intercept	−3.82 (0.92)	0
(LACE score)^2^	0.01 (0)	0.0001
(RBC zinc)^2^	0.01 (0)	0.0844

This table presents parameter estimates (with SE) and *P* values of terms in 4 logistic regression models with the 30-d death or urgent readmission as the outcome. Negative parameter estimates indicate an increased outcome risk as values decrease. Adjusted models include the LACE score [21]. Analyses were limited to people who survived their index hospitalization (*n* = 241). Plasma and RBC zinc concentrations were measured in micromoles per liter. LACE, length of stay, acuity of admission, comorbidities, emergency department use; .

## Discussion

The association of plasma and RBC zinc concentrations with important outcomes in hospitalized patients is unclear. This study prospectively recorded plasma and RBC zinc concentrations in a wide variety of patients early in their admission. By linking to population-based registries, we found that plasma zinc concentrations—but not RBC zinc concentrations—were significantly and negatively associated with long-term survival, even after adjusting for a validated hospitalized-patient survival index. Neither zinc measure was significantly associated with 30-d death or urgent readmission rates.

Our study yielded several novel findings. First, our data found in hospitalized patients an independent association between decreased plasma zinc concentrations and an increased risk of death of any cause. It is notable that significant differences in survival stratified by plasma zinc concentrations persisted even a year after the initial hospital admission and zinc measurement. Eleven previous studies have examined the association between plasma zinc and death risk in hospitalized patients [10]. These studies tended to involve critical care patients (8 studies), were small (median of 112 patients), had short time horizons (8 studies determined death status at 30 d or less), and used crude analytical methods (only 1 study [26] used survival analytical methods that adjusted for potential confounders). This study included patients outside of the critical care unit in whom zinc concentrations were measured early during their admission and followed them up for almost 2 y. We found that all-cause patient survival decreased significantly as plasma zinc concentrations decreased, even after adjusting for an accurate and validated death index. Second, RBC zinc concentrations were not associated with patient survival rates. To our knowledge, the relationship between RBC zinc concentrations and long-term patient outcomes has not been studied in the past. Third, neither plasma nor RBC zinc concentrations are significantly associated with 30-d death or urgent readmission risk rates.

Several factors should be kept in mind when interpreting our data. First, we found that the negative association between plasma zinc concentrations and patient survival remained after adjusting for a validated and accurate hospitalized-patient survival index. However, plasma zinc concentrations are strongly associated with other factors—such as hemoglobin concentrations and neutrophil-to-lymphocyte ratio [12]—that could act as confounders in the association between zinc concentrations and survival rates. This study could not adjust for these and other potentially important but unknown factors that could be relating zinc concentrations and survival rates. Second, although we found an independent association between plasma zinc concentrations and patient survival, we have not determined whether this association is actually causal. Ideally, this would be determined in a randomized interventional trial of hospitalized patients because zinc concentrations can be increased with an oral supplementation [27]. Third, our sample excluded patients who were unable to consent to the study, including those who were severely ill and patients with acute delirium or chronic cognitive issues. We are uncertain whether our findings would apply to these people. Fourth, we attempted to obtain fasting zinc measurements by procuring samples with morning blood workup because zinc concentrations can decrease postprandially [28,29]. However, it is unclear whether all samples were fasting measures owing to delays and logistical challenges within the hospital. Finally, this study may have been underpowered to detect a weaker underlying association between zinc concentrations and the secondary outcome of 30-d death/urgent readmission risk because these were short-term outcomes with more infrequent event rates compared with all-cause death rates.

In summary, we found that plasma zinc was significantly associated with patient survival even after adjusting for a validated expected death risk index. Neither plasma nor RBC zinc concentrations were significantly associated with 30-d death rates or urgent readmission risk. Future research is required to determine whether supplementing zinc in hospitalized patients changes patient survival.

## Funding

This study was supported by The Ottawa Hospital Academic Medical Organization.

## Author disclosures

All authors report no conflicts of interest.

## Declaration of interests

The authors declare the following financial interests/personal relationships which may be considered as potential competing interests:

Carl van Walraven reports financial support was provided by The Ottawa Hospital Academic Medical Association (TOHAMO).

## Data Availability

The data described in the manuscript, code book, and analytic code will not be made available because of privacy regulations.
