# Supplementary material for: The Independent Association of Plasma and Red Blood Cell Zinc Concentrations with Long-Term Outcomes of Hospitalized Patients
Source: Curr Dev Nutr. 2023 Mar 2;7(4):100062. doi: 10.1016/j.cdnut.2023.100062 (PMC10257222; doi:10.1016/j.cdnut.2023.100062)
Supplement: Multimedia component 1 [file mmc1.docx]

**SUPPLEMENTARY MATERIAL FOR:
THE INDEPENDENT ASSOCIATION BETWEEN PLASMA AND RED BLOOD CELL ZINC LEVELS WITH LONG-TERM OUTCOMES OF HOSPITALIZED PATIENTS**

**STEFAN RODIC ET. AL.**

**APPENDIX A:** Details regarding plasma and RBC zinc mesasurements.

**Plasma**

- 0.3 ml sample/QC/STD’s + 5.7 ml diluent (0.1% HNO3 +0.5% etoh +0.0125% triton X, 5ug/L Rhodium as ISTD)
- Samples were mixed and then ready for analysis
- Samples were measured in medium resolution ( ~ 4000)
- 6-pt standard curve was used
- Data were collected over 9 runs with 30 samples/peak
- Seronorms L1 and L2 were included 3 times per run (3 L1 and 3 L2 for a total of 6 QC samples spread out over the run)
- Seronorm info is provided here : <https://www.sero.no/products/seronorm-trace-elements-serum/#:~:text=Seronorm%E2%84%A2%20Trace%20Elements%20Serum%20is%20an%20accuracy%20control%20for,safety%2C%20veterinary%20and%20biological%20studies>.

**RBC**

- Packed RBC harvested according to your local protocol. Most collect in royal blue top tube, spin down and pour off plasma. Some will wash RBC with saline, although the impact of washing isn’t very much assuming you fill the tube with whole blood as per manufcturer
- We actually digest the RBC so getting internal RBC volume is actually pretty good this way and not as challenging as you thought, this turn proteins, lipids and DNA into CO2 NOx etc so you are only left with the liquid portion
- 0.5 ml Whole blood or ERC +0.6 ml HNO3 ( 67-70% ) placed in lightly capped tubes and place onto 108 degrees C hot block for 25 minutes
- Tubes cool to RT, then add 0.1 ml hydrogen peroxide, place back on hot block for 3 more minutes to finish digestion
- To digested sample, add 6 mL of diluent ( 0.75% etoh, 5 ug/L rhodium)
- Analyzed in medium resolution
- 9 runs 45 samples/peak
- 6pt standard curve

More information can be found here:

[https://www.sero.no/products/seronorm-trace-elements-whole-blood/](https://can01.safelinks.protection.outlook.com/?url=https%3A%2F%2Fwww.sero.no%2Fproducts%2Fseronorm-trace-elements-whole-blood%2F&data=05%7C01%7Ccvanwalraven%40toh.ca%7C665d84b6b304418a56b108db0537c43d%7C859b41b6130f4d13a6931ffec4e7cb5a%7C0%7C0%7C638109509295014597%7CUnknown%7CTWFpbGZsb3d8eyJWIjoiMC4wLjAwMDAiLCJQIjoiV2luMzIiLCJBTiI6Ik1haWwiLCJXVCI6Mn0%3D%7C3000%7C%7C%7C&sdata=Fu7LgaTRckLEmYwh2El8UXplcMUfwf0aZDb5vPaebms%3D&reserved=0)

**APPENDIX B:** Participant flow chart

**Patients Recruited**

314

No samples taken (N=25)

**Zinc samples ≤2 days**

289

Invalid samples due to insufficient blood volume or clotting (N=37);

**Plasma+RBC zinc samples**

252

\

Invalid health card number preventing linkage with population-based data (N=2)

**Patients Included**

250

**APPENDIX C:** Analyses examining for interaction between zinc levels and patient factors with study outcomes

| **INTERACTION VARIABLE** | **ZINC MEASURE** | **OUTCOME** | |
| --- | --- | --- | --- |
|  |  | **DEATH** | **30-DAY DEATH OR UNPLANNED READMISSION** |
| SEX | PLASMA | P=0.8199 | P=0.1052 |
|  | RBC | P=0.3424 | P=0.5551 |
| AGE | PLASMA | P=0.3904 | P=0.2541 |
|  | RBC | P=0.7593 | P=0.4454 |

This table presents the p-value for interaction terms added to the models presented in Table 2. None of the interactions meet standard criteria for statistical significance.
